# Transcription of Genes Involved in Sulfolipid and Polyacyltrehalose Biosynthesis of *Mycobacterium tuberculosis* in Experimental Latent Tuberculosis Infection

**DOI:** 10.1371/journal.pone.0058378

**Published:** 2013-03-05

**Authors:** Jimmy E. Rodríguez, Ana S. Ramírez, Laura P. Salas, Cecilia Helguera-Repetto, Jorge Gonzalez-y-Merchand, Carlos Y. Soto, Rogelio Hernández-Pando

**Affiliations:** 1 Departamento de Química, Facultad de Ciencias, Universidad Nacional de Colombia, Bogotá, Colombia; 2 Laboratorio de Microbiología Molecular, Escuela Nacional de Ciencias Biológicas, Instituto Politécnico Nacional, México D.F., México; 3 Sección de Patología Experimental, Instituto Nacional de Ciencias Médicas y Nutrición “Salvador Zubirán”, México D.F., México; University of Padova, Italy

## Abstract

The Influence of trehalose-based glycolipids in the virulence of *Mycobacterium tuberculosis* (*Mtb*) is recognised; however, the actual role of these cell-wall glycolipids in latent infection is unknown. As an initial approach, we determined by two-dimensional thin-layer chromatography the sulfolipid (SL) and diacyltrehalose/polyacyltrehalose (DAT/PAT) profile of the cell wall of hypoxic *Mtb*. Then, qRT-PCR was extensively conducted to determine the transcription profile of genes involved in the biosynthesis of these glycolipids in non-replicating persistent 1 (NRP1) and anaerobiosis (NRP2) models of hypoxia (Wayne model), and murine models of chronic and progressive pulmonary tuberculosis. A diminished content of SL and increased amounts of glycolipids with chromatographic profile similar to DAT were detected in *Mtb* grown in the NRP2 stage. A striking decrease in the transcription of *mmpL8* and *mmpL10* transporter genes and increased transcription of the *pks* (polyketidesynthase) genes involved in SL and DAT biosynthesis were detected in both the NRP2 stage and the murine model of chronic infection. All genes were found to be up-regulated in the progressive disease. These results suggest that SL production is diminished during latent infection and the DAT/PAT precursors can be accumulated inside tubercle bacilli and are possibly used in reactivation processes.

## Introduction

*Mycobacterium tuberculosis* (*Mtb*) is responsible for the greatest number of deaths by a bacterial pathogen worldwide [1]. *Mtb* can produce progressive disease or latent infection [2]; indeed, in high endemic areas, infection first occurs in childhood and is controlled in most cases. Only 10% of these primary infections lead to progressive disease [2], [3]; however, some bacilli remain in tissues in a non-replicating dormant or slowly replicating stage for the rest of the individual’s life. This latent tuberculosis (LTB) is clinically asymptomatic, and in countries with low or moderate endemicity, most active tuberculosis (TB) cases arise as a result of the reactivation of latent bacilli [2], [3]. It is estimated that one third of the world’s population carries latent *Mtb*, and millions of TB reactivation cases are predicted to occur in the coming years [4].

It is widely accepted that cell-mediated immunity, primarily by Th-1 cytokines and TNF-α, controls LTB and that dormant bacilli persist in granulomas under hostile conditions [5]–[7]. Experimental models of LTB have been performed *in vitro* to simulate the conditions faced by latent bacilli in granulomas, such as low-oxygen tension, starvation or acid pH, and *in vivo* using animal models to closely simulate the immunological response related to LTB in mammal tissues. The hypoxic “Wayne” model has been widely used as an *in vitro* model in which oxygen is gradually withdrawn, thereby inducing mycobacteria to enter into a non-replicative persistence (NRP) state [8]. *In vivo*, it is possible to induce progressive disease or latent infection by the intratracheal inoculation of a high or low infecting dose of *Mtb* in BALB/c or C57bl/DBA mice, respectively [9], [10]. This LTB model is reproducible and it is characterized by granuloma formation, high expression of TNFα, iNOS, IL-2 and IFNγ without tissue damage (pneumonia) displaying low and stable lung bacillary loads [9]. In contrast, progressive TB is produced by the intratracheal administration of high infecting dose that ensures bacilli proliferation, formation of granulomas with mild interstitial and perivascular inflammation raising maximal Th-1 protective response at day 21 of infection, followed by progressive bacilli burdens, in coexistence with tissue damage and emergence of Th-2 cells [9], [10].

**Figure 1:**
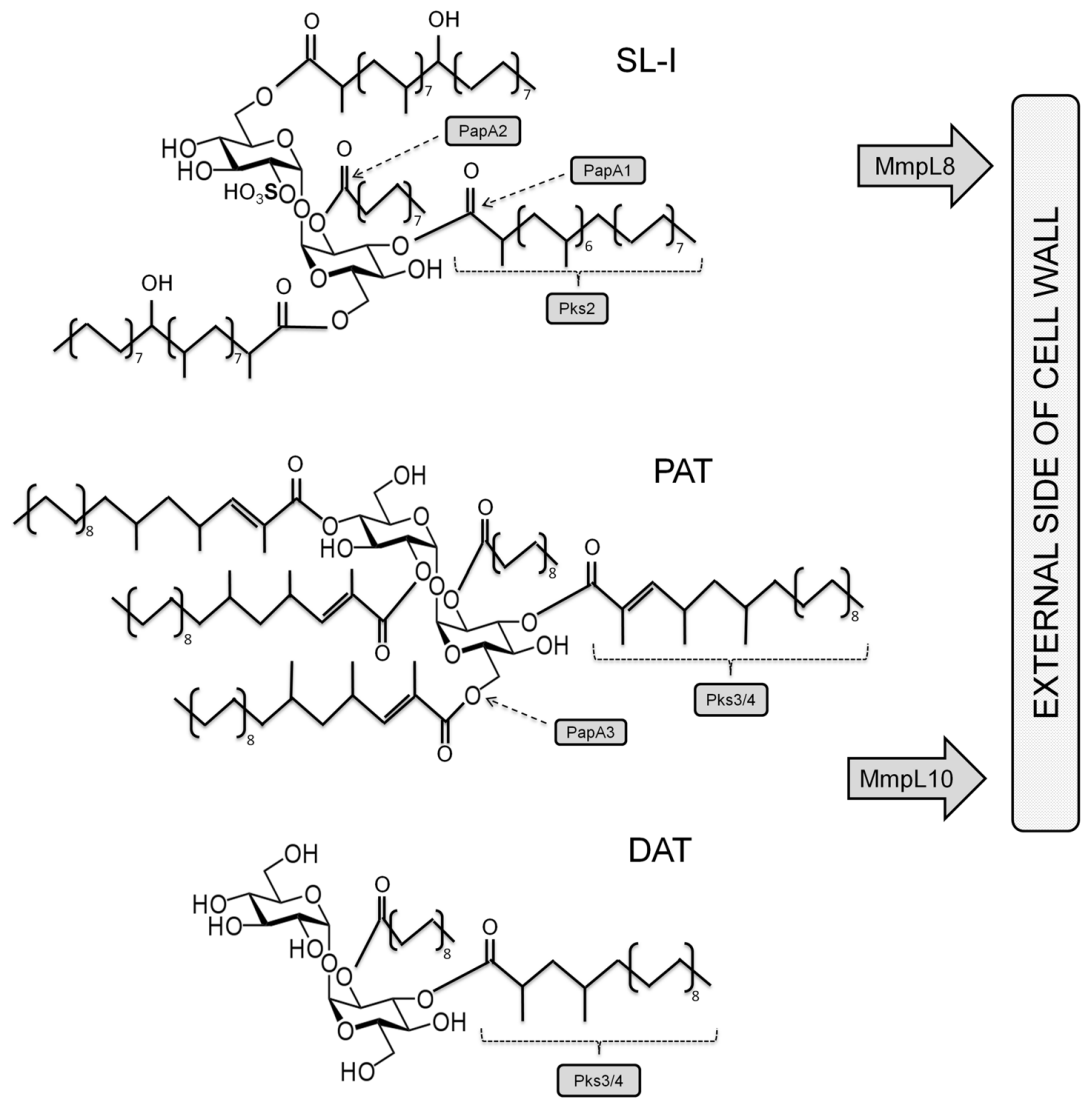
Structure of SL-I and DAT/PAT of *M. tuberculosis*. The trehalose core is sulphated in position 2′ and esterified with palmitic, multimethyl-branched phtioceranic and hydroxyphtioceranic acids in positions 2, 4, 6 and 6′ for SL-I biosynthesis; it is also esterified with stearic and multimethyl-branched mycolipenic acids in positions 2, 2′, 3′, 4 and 6′ for PAT, or with mycosanoic acids for DAT biosynthesis. The enzymatic actions of Pks: polyketidesynthase, PapA: acyltransferases and MmpL: mycobacterial membrane protein-large polyketide transporters are shown by dashed lines and arrows.

An important characteristic of latent bacilli is their gradual loss of acid-fastness associated with variations in the cell wall microstructure, suggesting alterations in lipid composition [6]. In addition, hypoxia induces the transcription of genes involved in the biosynthesis and production of some cell-wall glycolipids [11]–[14] and mycolic acids [11]. Trehalose-based glycolipids are significant constituents of the mycobacterial cell wall, with diacyltrehalose/polyacyltrehalose (DAT/PAT) and sulfolipid (SL) being important members of this group; the mechanisms of SL and DAT/PAT biosynthesis have been recently proposed [15]–[19]. Thus, the polyketide synthases (Pks) 2, 3 and 4 synthesise acyl chains that esterify hydroxyl groups of trehalose by the action of the PapA1, PapA2 and PapA3 acyltransferases (Figure 1). Subsequently, the MmpL8 and MmpL10 membrane-associated transporters export these synthesised lipids or their precursors to the external side of the cell wall.

The *Mtb* two-component system PhoP-PhoR positively regulates the synthesis of SL, DAT and PAT during bacilli phagocytosis; therefore, disruption of *phoP* in *Mtb* yields mutants lacking trehalose-based glycolipids, which is also observed in the attenuated *Mtb* strain, H37Ra [20]. Regarding their function in the host, SL inhibits mitochondrial oxidative phosphorylation and blocks phagosome-lysosome fusion in macrophages; SL also modulates the oxidative response and the secretion of anti-inflammatory cytokines (IL-10 and IL-13) by human monocytes and neutrophils [21]–[23], and accumulation of SL precursors can stimulate human CD1b-restricted T cells [23].

Several studies have shown that *Mtb* H37Rv knock-out mutants of *pks2* are defective in SL biosynthesis; however, these strains do not show a significant decrease in persistence or pathogenicity in mice or macrophages [22]. *Mtb mmpL8* knock-out mutants produce diacylated forms of SL and are less virulent in mice. *In vitro* studies have shown that mycolipenic and mycoseric acids, the acyl substituents in DAT and PAT, inhibit leukocyte migration, but mutants that do not synthesise mycolipenates and mycosanoates do not show virulence alteration in macrophages or mice [21], [22]. The actual role of trehalose-based glycolipids in LTB is completely unknown. Thus, the aim of the present study was to evaluate the transcriptional behaviour of genes involved in the SL and DAT/PAT biosynthesis in *in vitro* and *in vivo* experimental models of LTB. We observed that the genes involved in SL and PAT biosynthesis of *Mtb* are differentially expressed in experimental progressive and LTB infection.

## Materials and Methods

### Ethics Statement

All the animal work was done according to the guidelines and approval of the Ethical Committee for Experimentation in Animals of the National Institute of Medical Sciences and Nutrition in Mexico (CINVA), permit number: 224. All surgery was performed under sevofluorane anaesthesia, and all efforts were made to minimize suffering.

### Mycobacterial Strains and Growth Conditions

*Mtb* H37Rv (ATCC 27294, Rockville, MD, USA) was used in this study. Mycobacteria were cultured at 37°C in Dubos-ADC (Difco) until the exponential phase of growth was reached (standard conditions). The cultures were then subjected to non-replicative persistence 1 (NRP1) and non-replicative persistence 2 (NRP2) or anaerobiosis stages as described by Wayne and Hayes [8]. A parallel culture supplemented with methylene-blue (1.5 µg mL^−1^) was used as an indicator of oxygen depletion. Mycobacteria grown in standard and hypoxic (NRP1 and NRP2) conditions were used for RNA isolation and lipid analysis. All determinations were performed at least in duplicate.

### Lipid Extraction and Analysis by TLC

Glycolipids were extracted as previously described [24] from oxygenated mycobacteria harvested in exponential phase of growth and NRP2 stage. The SL and DAT content was analysed on 20×20 Silica Gel 60 thin-layer chromatography (TLC) plates (Merck, Germany) by two-dimensional TLC [25]. Mycolic acids were analysed as described previously [26]. Carbohydrate-containing compounds were visualised by spraying TLC plates with 1% anthrone (Sigma, USA) in H_2_SO_4_, followed by heating at 120°C. Methyl mycolates were visualised using 10% molybdophosphoric acid (Merck, Germany) in ethyl alcohol (w/v) and heating at 120°C. *Mtb* H37Rv (ATCC 27294) SL-I, DAT and CF standards were developed and visualized in parallel experiments.

### Neutral-red Test

*Mtb* cells were placed in screw-cap tubes containing 50% (v/v) aqueous methanol and incubated for 1 h at 37°C. The fluid was then removed, 0.002% neutral red in Tris-HCl buffer at pH 9.8 was added, and the tubes were kept at room temperature for 24 h [27].

### Experimental Models of Latent Mycobacterial Infection and Progressive Pulmonary Tuberculosis in Mice

The models of latent and progressive TB in mice have been described elsewhere [9], [10]. The *Mtb* strain H37Rv was grown until an OD_600_ of 0.4–0.8 was reached. Bacilli were harvested, adjusted to 1×10^3^ bacteria/100 µL in phosphate buffered saline (PBS), aliquoted and maintained at −70°C until use. The viability of the bacteria was checked before infection. Induction of chronic infection with a long-lasting stable infection similar to LTB was performed with 6–8-week-old female B6D2F1 (C57BL/6J×DBA2/J) mice (Jackson Laboratories Bar Harbor, ME, USA). Mice were anesthetised with sevofluorane and inoculated intratracheally with 1×10^3^ viable bacilli in 100 µL of PBS. In addition, a group of mice with stable latent TB after 7 months of infection were supplemented with corticosterone (3 mg mL^−1^) dissolved in their drinking water to induce reactivation. Groups of three mice were killed by exsanguination at 1, 3 and 5 months after intratracheal infection and 20 days after corticosterone supplementation.

To induce progressive pulmonary TB, 6–8-week-old male BALB/c mice were anesthetised with sevofluorane and inoculated intratracheally with 2.5×10^5^ bacilli in 100 µL of PBS (treatment of bacteria was similar to the LTB model). Groups of three mice were killed by exsanguination at 1, 28 and 120 days after intratracheal infection. All the animal work was done according to the guidelines and approval of the Ethical Committee for Experimentation in Animals of the National Institute of Medical Sciences and Nutrition in Mexico (CINVA), permit number: 224. All surgery was performed under sevofluorane anaesthesia, and all efforts were made to minimize suffering.

### RNA Isolation and cDNA Synthesis

RNA from *Mtb* was extracted using TRIzol reagent (Invitrogen, USA) as described elsewhere [28], [29]. For *in vitro* analysis, total RNA from standard *Mtb* cultures was isolated in the exponential and stationary phases of growth; for hypoxic conditions, RNA was isolated from cultures in NRP1 (after 5 days of exposure to hypoxia) and NRP2 (after 11 days of exposure to hypoxia) stages of Wayne’s model of dormancy. Total RNA was dissolved in DEPC- treated H_2_O and stored at −70°C. A previously described technique was used to determine mycobacterial gene expression by quantitative real-time PCR (qRT-PCR) after the isolation of mycobacterial RNA from tissues [28]. For each time point of the *in vivo* infection TB models, lungs from mice were perfused with 1 mL of lysis buffer (6 M guanidinium chloride, 0.1% Tween 80, 10 mM EDTA and 1 mM mercaptoethanol) and frozen by liquid nitrogen immersion. These tissues were kept at −70°C until use. Lungs were disrupted in o-ring tubes containing 2 ml of lysis buffer and zirconium-silica beads (710–1180 and 150–212 µm, Sigma) in Fast-Prep equipment (Thermo, Germany). Total RNA from these homogenates was isolated with the same methodology used for mycobacteria in culture. cDNA from all samples was prepared using 2 µg of RNA, random hexamers (0.5 µg/µl) and Super Script® reverse transcriptase (20 U µL^−1^, Invitrogen).

### Quantitative Real-Time PCR (qRT-PCR)

The genes involved in SL biosynthesis (*pks2*, *papA1*, *papA2* and *mmpL8*) and DAT/PAT biosynthesis (*pks3*, *pks4*, *papA3*, *mmpL10*), together with the *phoP* and *rfpB* genes, were quantified by qRT-PCR using specific primers (Table 1). Total mRNA transcription was normalised to the mean value of 16S rRNA (*rrs*) gene expression [30]. Quantitative differences in transcription among growth phases and periods of *in vivo* infection were analysed with logarithmic graphs of each gene (reported as copies of the gene/µg RNA). Fluorescence was quantified using LC FastStart DNA Master SYBR Green I (Roche) and the LightCycler® 1.5 system (Roche). To validate qRT-PCR assays and determine their efficiency, serial dilutions of genomic DNA of *Mtb* H37Rv were tested. Quantification was performed four times for each condition, and results were analysed using a calibration curve for which the regression value was close to 1 and the efficiency was close to 2.

**Table 1:** Primers for qRT-PCR used in this study.

Primer	Sequence (5′–3′)	Reference
*pks2*F	CGGTGACCCCATTGAATAT	This study
*pks2*R	CACCATGTTTCAGAGCGAGA	This study
*papA1*F	GATGCTAATGGACGAGCAG	This study
*papA2*R	CGTCACTGTGGTCTGATGCT	This study
*mmpL8*F	GGCGGGGTTCTATATTCC	This study
*mmpL8*R	GGATTCAGGTCGGTTTGTAT	This study
*pks3*F	GATCAGCTGGCTGAGATTG	This study
*pks3*R	GCCGCTGGTTCTGATAC	This study
*pks4*F	TATGGGGTACGTCCTGGTG	This study
*papA3*F	GCGATGTTCCGGTCAGTT	This study
*papA3*R	GTACGCCGGATTATTTGTCC	This study
*mmpL10*F	GGCTTGACTCTGTTGTCGC	This study
*mmpL10*R	GATTTCAGCGAGCGGACTA	This study
*phoP*F	TCTCGACCACGTTTGGCGCT	This study
*phoP*R	CCCGCAGTACGTAGCCCACC	This study
*rpfB*F	CCGCAATCGGATCAAGAA	[30]
*rpfB*R	CGACCTCCCGGCTCAT	[30]
16SrRNAF	ATGACGGCCTTCGGGTTGTAA	[30]
16SrRNAR	CGGCTGCTGGCACGTAGTTG	[30]

### Statistics

For each experiment, differences among experimental data were compared using Two-Way ANOVA, Tukey’s multiple-comparison procedure. Data were accepted as significantly different if *P*<0.05.

## Results

### Hypoxia Induces Change in the Trehalose-based Glycolipid Profile of *Mycobacterium tuberculosis* H37Rv

**Figure 2:**
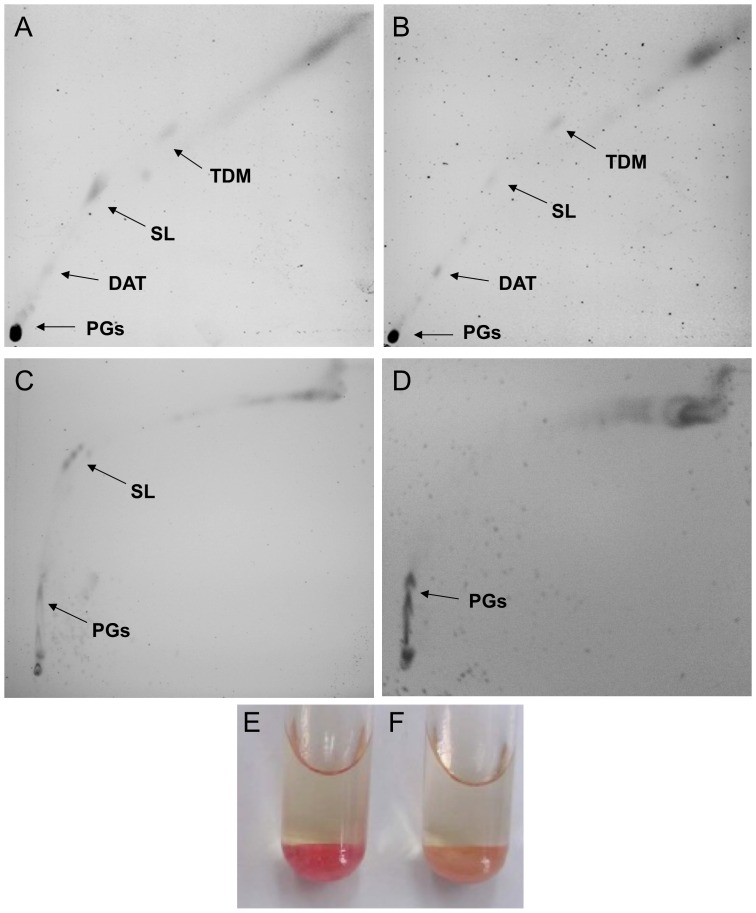
Trehalose-derived glycolipid profile and neutral red staining of *M. tuberculosis* grown in NRP2 stage. 2D-TLC analysis *of Mtb* H37Rv grown under oxygen-replete (A and C) and NRP2 (B and D) conditions. Crude extracts were resolved using the two-solvent system: chloroform:methanol:water (60∶12:1, v/v) for the first direction and chloroform:methanol:water (75∶11:1, v/v) for the second direction. For resolving polar glycolipids (PGs) accumulated in the inoculation point, the elusion time in C and D was prolonged for 30 min after the resolving system reached the edge of the TLC plate. The neutral red staining observed for *M. tuberculosis* H37Rv cultured in aerobic (E) and NRP2 stages (F) is shown. TDM, trehalose dimycolate; SL, sulfolipid; DAT, diacyltrehalose; PGs, polar glycolipids.

Cultures of *Mtb* obtained under hypoxic conditions produced more dispersed mycobacteria, indicating a possible alteration of the lipid content on the bacterial surface. Non-covalently attached lipids were extracted from *Mtb* grown in oxygen-supplied and NRP2 conditions. As an initial approach, analysis of crude lipid extracts by TLC revealed similar profiles of phospholipids and phosphatidylinositol mannoside (PIM) under both conditions. Accumulation of triacylglicerol (TAG) in NRP2 was also observed (data not shown). The glycolipid TLC profile of *Mtb* H37Rv in NRP1 was similar to that observed in bacilli grown under standard conditions (data not shown). According to the purified glycolipid standards used in the 2D-TLC analysis, trehalose-based glycolipids in NRP2 showed deficient production of SL, an increased DAT content, and the accumulation of polar glycolipids with chromatographic behaviour similar to PAT (Figure 2).

With regard to the mycolic acid content, oxygenated and hypoxic (NRP2) cells showed similar pattern of mycolates; the characteristic α (I), methoxy (III) and keto-mycolates (IV) for *Mtb* were detected under both growth conditions (data not shown). Finally, *M. tuberculosis* in NRP2 did not become red when cells were subjected to neutral red staining (Figure 2F).

### *Mycobacterium tuberculosis* Displays a Differential Transcription of *mmpL* and *pks* Genes in the NRP2 Stage

RNA was isolated from two replicas of standard (oxygenated) *Mtb* H37Rv cultures in the exponential and stationary phases of growth and hypoxic (NRP1 and NRP2) conditions to quantify the transcription pattern of the selected genes by qRT-PCR. These mycobacterial genes related to glycolipids production were chosen according the gene clusters previously proposed for the SL-I and DAT/PAT biosynthetic pathways (See Figure 1) [17]. At the present time, the biosynthetic pathway of DAT is not well-known.

**Figure 3:**
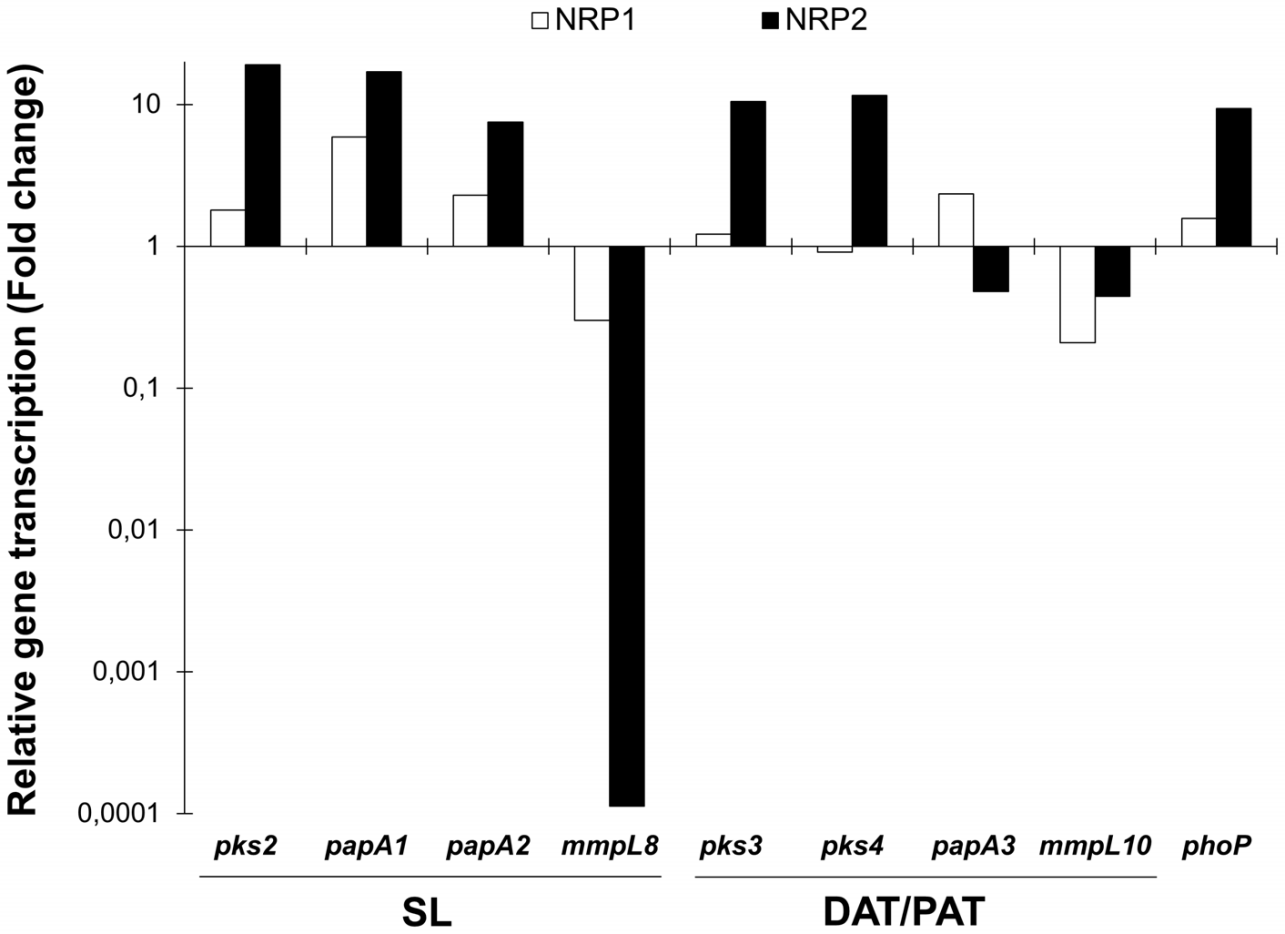
*In vitro* transcription of genes in *M. tuberculosis* H37Rv during the NRP1 and NRP2 stages. The relative quantification is expressed as the ratio of NRP1 and NRP2/transcription of exponential-oxygenated phase. The presented data have statistically significant differences compared to exponential phase values (*P*<0.05).

When compared with bacteria in the exponential phase, oxygenated bacilli grown until the stationary phase exhibited increased transcription of *papA1* and *papA2,* both involved in the SL biosynthesis, and decreased transcription of *mmpL10* that codify for the transporter in the DAT/PAT biosynthesis (Figure S1). Under hypoxic conditions, the highest differences in transcription were observed in NRP2 in comparison to oxygenated bacilli in the exponential phase. With respect to the SL biosynthesis, the relative quantification showed that the transcription of *pks2*, *papA1* and *papA2* genes was 19-, 17- and 8-fold higher in NRP2 respectively. Conversely, the transcription rate of the *mmpL8* gene was 3-fold lower in NRP1 and extremely low (more than 8×10^3^-fold) in NRP2 (Figure 3). With regard to the DAT/PAT biosynthesis, the transcription of *pks3/pks4* was 10- to 11-fold higher, respectively, in NRP2. The transcription rate of the *mmpL10* gene was 5- and 2-fold lower in NRP1 and NRP2, respectively. Finally, the transcription of the *phoP* regulator was 2- and 10-fold higher in NRP1 and NRP2, respectively (Figure 3).

### Differential Transcription of Genes Involved in SL and DAT/PAT Biosynthesis in Murine Models of Progressive and Latent Tuberculosis Infection

**Figure 4:**
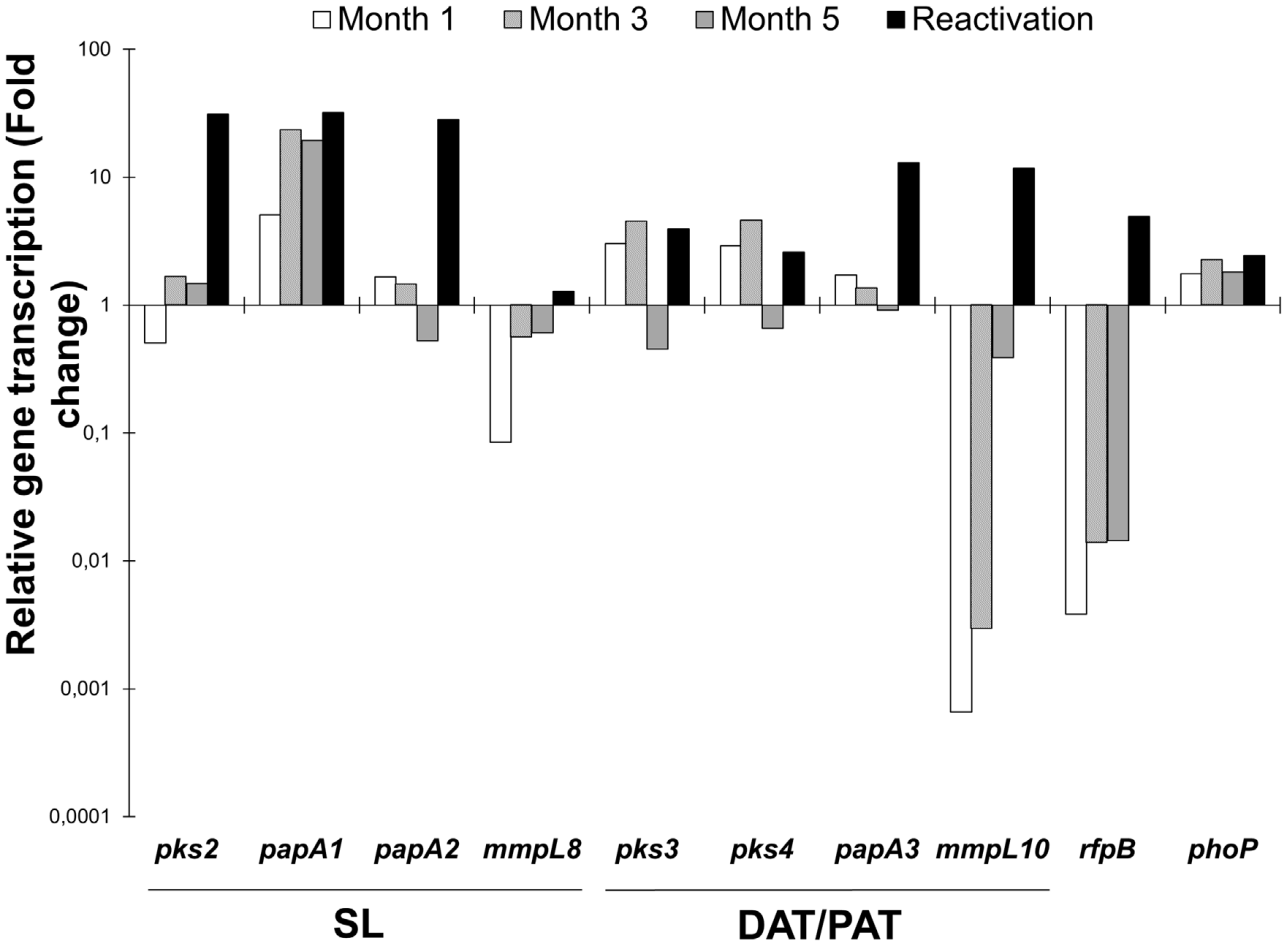
*In vivo* transcription of genes in *M. tuberculosis* H37Rv during long-lasting TB infection in mice. The relative quantification is expressed as the ratio of infection at months 1, 3, 5 and reactivation/transcription at day 28 of progressive infection TB. The presented data have statistically significant differences compared to transcription values at day 28 of progressive infection TB (*P*<0.05).

In the chronic infection model that is similar to LTB, bacilli loads were constant (nearly 1×10^5^, data not shown). For relative quantification, the transcription level of the genes was compared with the transcription level on day 28 of the progressive disease. The transcription of some genes involved in the biosynthesis of SL and PAT (see Figure 1) increased during the course of experimental LTB, particularly *mmpL8* and *mmpL10*; however, the *pks3/pks4* genes exhibited slightly diminished transcription at month 5 in the LTB model (Figure 4). After LTB reactivation, all the studied genes exhibited significantly increased transcription. Reactivation was confirmed by the increased activity of the control gene *rfpB* (Figure 4), which was associated with high bacilli loads in the lungs. The transcription of *phoP* was always high and constant during the course of latent infection, reactivation and progressive disease (Figure 4).

**Figure 5:**
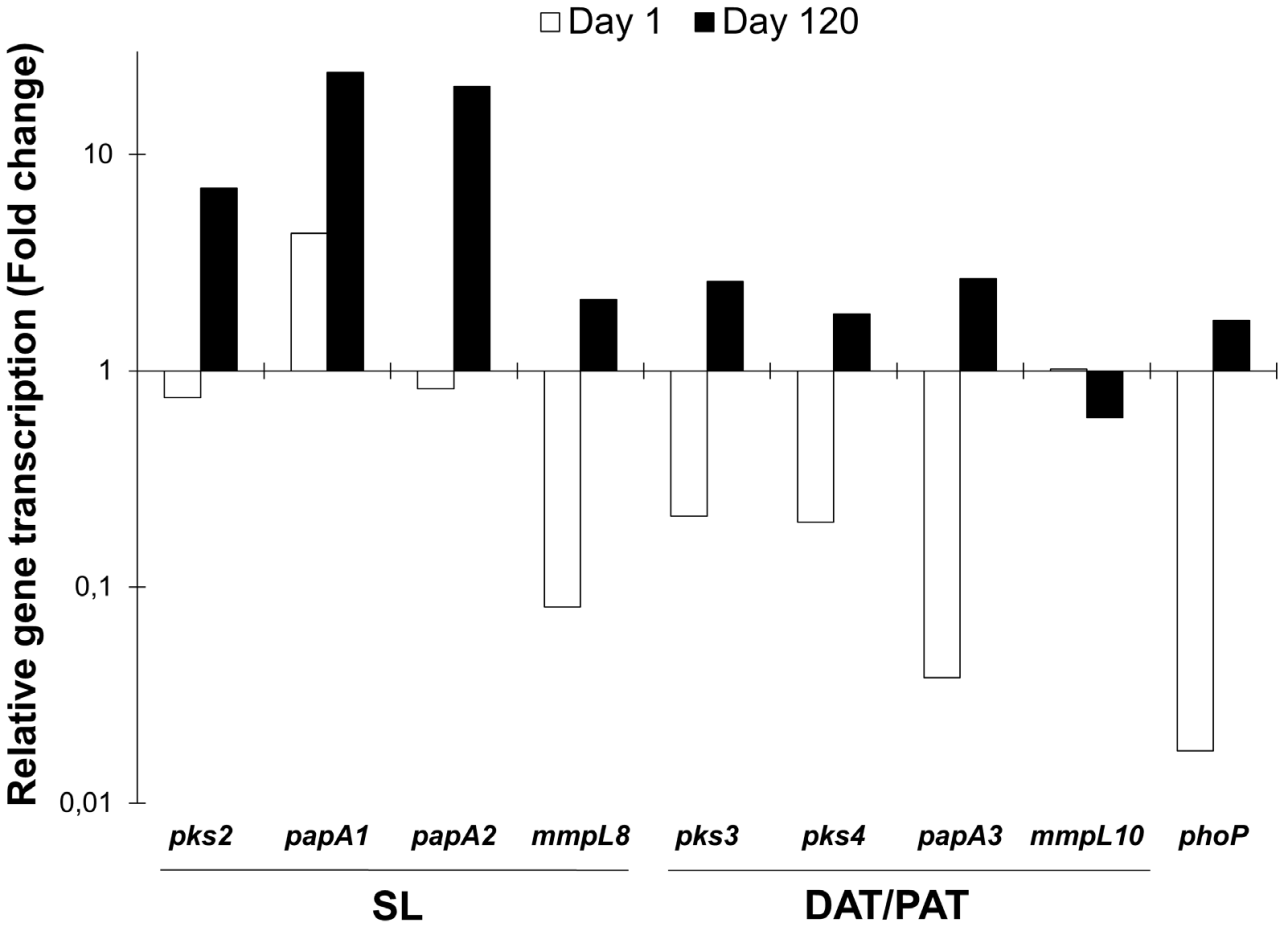
*In vivo* transcription of genes of *M. tuberculosis* H37Rv during progressive tuberculosis in mice. The relative quantification is expressed as the ratio of infection at days 1 and 120/transcription at day 28 of progressive infection TB. The presented data have statistically significant differences compared to transcription values at day 28 of progressive infection TB (*P*<0.05).

Regarding the progressive TB model, lung bacilli load determinations by CFU quantification showed that the initial bacilli load (2.5×10^5^) increased to 9.5×10^6^ at day 28 and 1.4×10^7^ at day 120 (data not shown). The transcription of most the selected genes strongly increased during the course of progressive infection (Figure 5). On day 1, the relative transcription of *mmpL8*, *papA3, pks3/pks4* and *phoP* was considerably less than for the other genes (2- to 60-fold lower); however, transcription of these genes, except *mmpL10*, increased considerably by day 120 (Figure 5). The values of relative quantification were based on absolute normalised quantification values showed in figures S2 and S3.

## Discussion

Because trehalose-based glycolipids such as SL and PAT are only present in virulent *Mtb* strains and therefore potentially play a role in virulence, recent studies have focused on establishing the actual function of this kind of glycolipid in TB pathogenesis [15]–[19]; however, their role in LTB remains unknown. A high bacterial load is necessary for a complete mycobacterial lipid constitution analysis, which is impossible to obtain from infected tissues. We therefore used the *in vitro* Wayne model to analyse the SL and DAT profile of the hypoxic *Mtb* cell wall. Although *in vitro* models do not completely reproduce the conditions of LTB, our results using the Wayne model showed previously unidentified alterations to the trehalose-based glycolipid composition of hypoxic *Mtb*. The murine model of chronic infection similar to LTB was used to compare with the *in vitro* lipid profile at the transcription level.

The TAG accumulation observed in hypoxic *Mtb* cells provided evidence that the oxygen-depletion model we employed resembled dormant-like bacilli [31]. *Mtb* under both, aerobic and NRP2 conditions, showed similar mycolate content, which is contrary to earlier studies that suggested that hypoxia is an important factor that induces the expression of proteins involved in mycolic acid biosynthesis [11]. Thus, the changes in the cell wall lipids that we observed in NRP2 cultures might be at the level of non-covalently attached glycolipids. The observation that hypoxic *Mtb* H37Rv results in negative neutral red staining suggests impaired production of some methyl-branched glycolipids and a possible reorganisation of the mycobacterial cell wall [20], [32]. SL, a methyl-branched glycolipid, is clearly decreased (Figure 2) in NRP2 stage**,** that contribute to the negative neutral red staining [32]. This behaviour is also observed in *phoP* mutants that are deficient in SL production [20].

**Figure 6:**
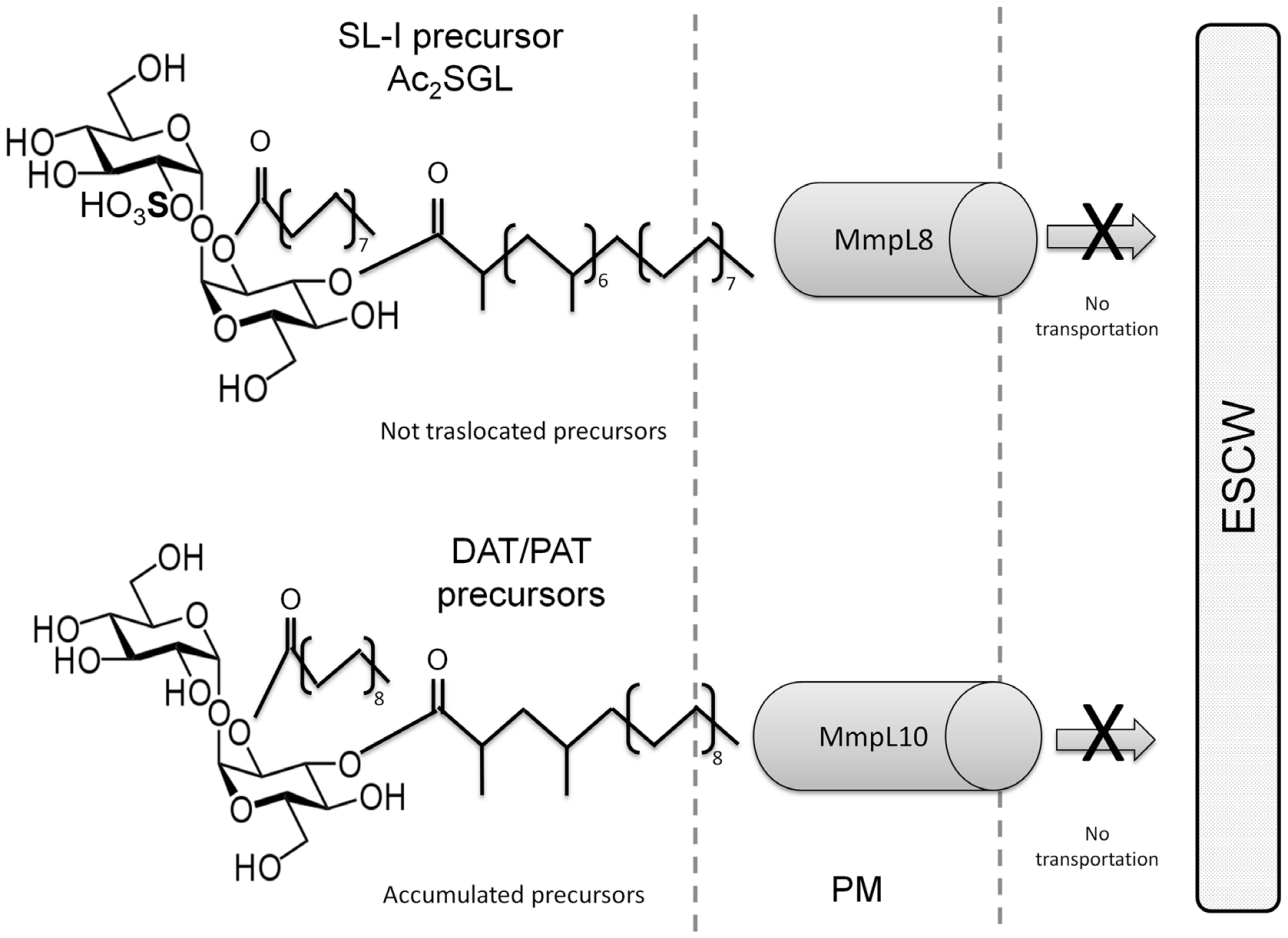
Model showing accumulation of DAT and SL/PAT precursors in latent *M. tuberculosis*. The diminished transcription of *mmpL8* and *mmpL10* might avoid the complete biosynthesis of trehalose-based glycolipids and the subsequent accumulation of precursors as polar glycolipids and DAT in the plasma membrane (See discussion for details). Plasma membrane (PM); external side of cell wall (ESCW).

The transcriptional profile observed in hypoxia of genes involved in the SL and DAT/PAT biosynthesis may be produced as a response to the metabolic stress generated by gradual oxygen depletion. As it can be deduced from *Mtb mmpL8* knock-out [21], [22], the significantly low transcription of *mmpL8* avoids SL translocation to the mycobacterial cell wall and the subsequent complete biosynthesis of this glycolipid, which may partially explain the very low SL content in crude lipid extracts of NRP2-stage *Mtb* (Figure 2 and 6). In addition, the high *pks2*, *papA1* and *papA2* transcription in NRP2 suggests over-production of the hydroxyphtioceranic and phtioceranic acids that bind trehalose and induce the intracellular synthesis of diacylated sulfoglycolipids (Ac_2_SGL), precursor glycolipids that are more polar than the completely assembled SL [33], [34]; therefore, it is probably accumulated as a polar glycolipid.

The increase of *pks3/pks4* transcription during hypoxia might induce the intracellular accumulation of mycolipenic and mycolipanoic acids. As observed in SL biosynthesis, the reduction of *mmpL10* transcription probably avoids the complete PAT biosynthesis and DAT translocation to the cell wall (Figure 6), which partially explains the DAT and polar glycolipids accumulation observed in lipid extracts from *Mtb* under NRP2-stage (Figure 2B); however, we cannot ruled out the possibility that other glycolipids can co-migrate with DAT in our chromatography study. Dormant-like mycobacteria use alternative carbon sources as fatty acids and cholesterol [35], [36]. Under hostile conditions, *Mtb* up-regulates the WhiB3 protein, which maintains redox homeostasis by regulating the biosynthesis of SL, TAG, PAT and phtiocerol dimycocerosate (PDIM) to reduce the metabolic stress generated by fatty acid catabolism under oxygen-limited conditions [37]. Consequently, hydroxyphtioceranic, phtioceranic, mycolipenic and mycolipanoic acids, together with SL and PAT precursors, can be accumulated in NRP2; in this sense, an increased expression of *papA2* and *papA1* may be correlated with the accumulation of lipid intermediates used as energy source substrates [31], [35].

Because the Wayne model does not establish the direct interaction of persistent mycobacteria with host cells, the present study was complemented with analysis of the transcriptional profile of SL and DAT/PAT using murine models. In the model of progressive TB, the low transcription of mycobacterial genes on day 1 is predictable because *Mtb* is in the process of adaptation. It appears that lipids such as SL and DAT/PAT are more relevant during late disease, i.e., after 28 days of infection, when in this model, there is a decrease in the protective Th-1 immune response, high bacilli loads and high transcription levels of genes involved in SL and PAT biosynthesis are observed (Figure 5 and S3). Thus, it seems that the biological role of these glycolipids is more relevant during advanced disease when there is high bacilli proliferation and tissue damage (day 120) [38], [39].

In the case of chronic infection similar to LTB, the gene transcriptional profile also shows that SL and PAT synthesis is incomplete, similar to that of the Wayne model of hypoxia. When confined in granulomas for a long time, *Mtb* has to face a predominant Th-1 immune response and high expression of TNF-α; therefore, biosynthesis of SL and PAT precursors may be associated with mechanisms of immune evasion. Furthermore, the inhibition of leukocyte migration by mycolipenic and mycolipanoic acid *in vitro* has been previously observed [22]. Thus, reduction in trehalose-based glycolipids production in LTB could be attributable to adaptation of tubercle bacilli to the hostile environment of granulomas; precursors of these glycolipids are may be preferentially used as a reserve energy and carbon source, inducing an increase in the transcription of *pks* and *papA* genes, as we observed [31], [35], [38]. *mmpL8* and *mmpL10* exhibit a tendency to increase as the course of long lasting infection progresses. This behaviour suggests that mycobacteria have a differential production of some trehalose-based glycolipids in the initial latent infection [8], [35] as part of an adaptive process as seen in the *in vitro* NRP2 stage. In the reactivation stage, the transcriptional profile of genes is similar to that on day 120 of progressive infection in mice, suggesting that SL and PAT are involved in anti-inflammatory process related to active TB, such as pneumonic lesions and other tissue damage that are also promoted by the activity of the *rpfB* reactivation promoter [40].

As expected, *phoP* shows a high transcription rate in hypoxia (NRP1 and NRP2) and during the entire process of latent infection, this observation was in agreement with the increase of the *pks* genes transcription [41] and the subsequent accumulation of polar precursors. This behaviour is important because the virulence regulator *phoP* induces transcription of the global latency regulator Rv3133/*dos*R, together with the genes responsible for the initial and enduring hypoxic response and genes involved in the biosynthesis of the trehalose-based glycolipids that are correlated with *Mtb* virulence [42], [43].

In conclusion, the results obtained in this work suggest that LTB produces changes in trehalose-based glycolipid and long-chain fatty acid production that probably affect *Mtb* virulence. It is possible that latent *Mtb* uses SL and PAT precursors only as a metabolic adaptation to the granuloma environment, while in late active TB, these glycolipids are over-produced, likely because they play a role in virulence.

## Supporting Information

Figure S1 ***In vitro*** **transcription of genes in oxygenated and hypoxic** ***M. tuberculosis*** **H37Rv.** Transcription was measured during the exponential and stationary phases of growth and the non-replicative persistence 1 and 2 (NRP1 and NRP2) stages of hypoxia. The transcription of genes was normalised to the mean value of 16SrRNA. Asterisks indicate statistically significant differences compared to exponential phase values (*P*<0.05).
(TIF)

Figure S2 ***In vivo*** **transcription of genes in** ***M. tuberculosis*** **H37Rv during long-lasting TB infection in mice.** The transcription of genes was normalised to the mean value of 16SrRNA. Asterisks indicate statistically significant differences compared to transcription values at day 28 of progressive infection TB (*P*<0.05).
(TIF)

Figure S3 ***In vivo*** **transcription of genes in** ***M. tuberculosis*** **H37Rv during progressive tuberculosis in mice.** The transcription of genes was normalised to the mean value of 16SrRNA. Asterisks indicate statistically significant differences compared to transcription values at day 28 of progressive infection TB (*P*<0.05).
(TIF)
